# Preemptive Frozen Elephant Trunk Technique for Hemostatic Use in Total Arch Replacement

**DOI:** 10.1016/j.atssr.2024.12.015

**Published:** 2025-01-10

**Authors:** Takahiro Ozeki, Yoshiyuki Tokuda, Yuji Narita, Masato Mutsuga

**Affiliations:** 1 Department of Cardiac Surgery, Nagoya University Graduate School of Medicine, Nagoya, Aichi, Japan

## Abstract

The frozen elephant trunk technique is now a fundamental method of total aortic arch replacement. However, in adults, it is sometimes necessary to perform distal aortic anastomosis on the deep side in the case of infected aortic arch aneurysm or coarctation surgery. We herein report 3 cases (2 infected aortic arch aneurysms and 1 aortic coarctation) in which our novel technique was used to secure hemostasis with a preoperatively sized frozen elephant trunk inserted into the distal anastomosis under median sternotomy alone.

The frozen elephant trunk (FET) technique has been widely applied in treatment of dissecting aortic aneurysm. In the FET technique, the distal aortic arch aneurysm is sealed for total aortic arch replacement instead of anastomosing the distal part or inserting into the true lumen to close the entry that connects to the false lumen. However, it is sometimes necessary to perform distal aortic anastomosis on the deep side. It is crucial to secure hemostasis after the anastomosis. The L-incision technique or anterolateral partial sternotomy technique is applied to manage deep distal-side anastomosis. We herein report a novel technique for securing hemostasis with insertion of a preoperatively sized FET into the distal anastomosis under median sternotomy alone.

## Technique

**Figure 1:**
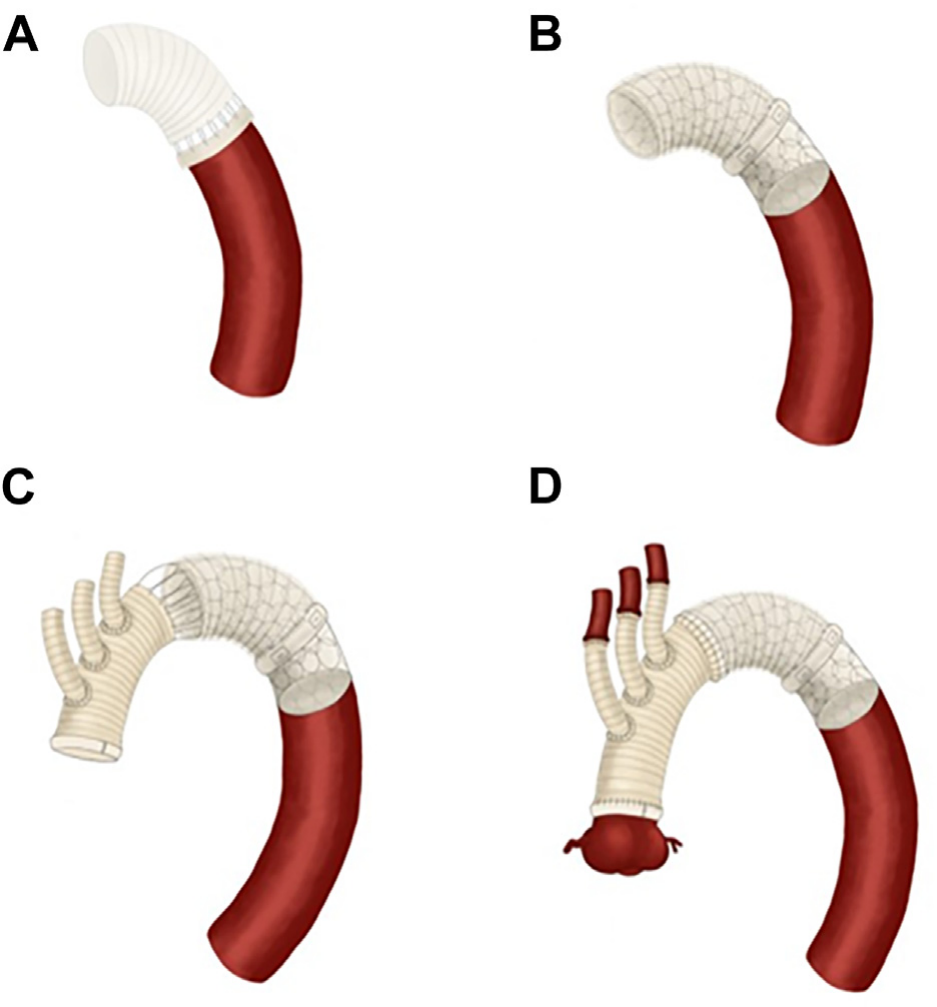
Implementation of preemptive frozen elephant trunk (FET). (A) Distal anastomosis to the descending aorta. (B) Insertion of FET with the distal tip approximately 3 to 4 cm distal to the anastomosis. (C) Anastomosed 4-branch artificial vessel and component of FET and unbranched artificial vessel that has already been anastomosed to the native vessel. (D) The proximal and branch anastomoses. All patients underwent the procedure in the same order.

The operation was performed through standard median sternotomy in all cases. The patient was cooled to a nasopharyngeal temperature of 25 °C by systemic cannulation into the proximal side of the ascending aorta with superior vena cava and inferior vena cava drainage. The systemic perfusion was terminated at the target temperature, and antegrade selective cerebral perfusion by direct cannulation was started. The descending aorta was dissected and anastomosed with the unbranched artificial vessel (Figure 1A). A FET, the size of which was estimated preoperatively on the basis of the measured diameter of the native descending aorta, was then inserted antegrade with the distal tip approximately 3 to 4 cm distal to the anastomosis (Figure 1B). The single tube of artificial vessel and FET were sutured with a 4-branch artificial vessel in 1 stage (Figure 1C). Lower body circulation was restarted, and the aortic clamp was released after the proximal anastomosis was performed. Anastomosis with each of the cervical branches was then completed (Figure 1D).

### Patient 1

**Figure 2:**
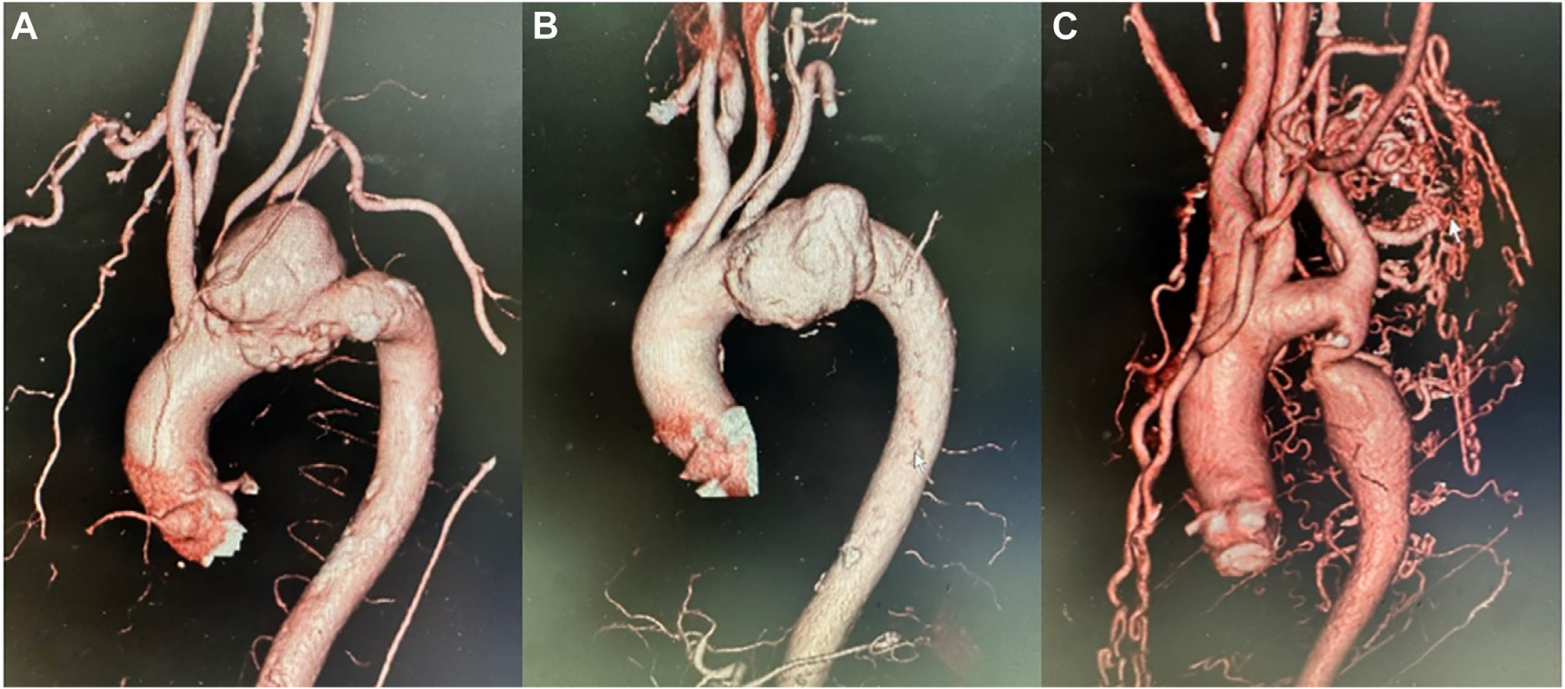
Preoperative computed tomography image of each patient. (A, B) Patients 1 and 2 with infectious aortic aneurysms. The suspected infectious aortic aneurysms are located at the distal arch. (C) Patient 3 with aortic coarctation.

A 73-year-old woman was referred to our department with impending rupture of a distal arch aneurysm. Computed tomography showed a saccular aneurysm with a diameter of 68 mm in the aortic arch. An infected aneurysm was suspected (Figure 2A).

Because of multiple adhesions from a previous thyroid gland resection, we performed distal anastomosis with a 24-mm unbranched section of artificial vessel by the inclusion technique and inserted a 27 × 120-mm FET. The patient was difficult to extubate because of general weakness, and tracheostomy was performed. She was transferred to another hospital for rehabilitation after removal of the tracheal cannula on postoperative day 91.

### Patient 2

A 74-year-old man was referred to our department with impending rupture of a distal arch aneurysm that was thought to be infected on the basis of high C-reactive protein level and rapid enlargement of the aneurysm (Figure 2B). After resection of the entire aneurysmal wall, a 28-mm unbranched section of artificial vessel immersed in rifampicin was anastomosed peripherally, and a 29 × 120-mm FET was inserted. There were no major complications, and the patient was discharged home on postoperative day 35.

### Patient 3

A 39-year-old woman was referred to our department with aortic coarctation (Figure 2C). Multiple adhesions were present because of surgery for patent ductus arteriosus at the age of 5 years. The working area was narrow, visibility was poor because of blood backflow, and the aortic wall was fragile. A 26-mm unbranched section of artificial vessel was anastomosed to the descending aorta and a 29 × 90-mm FET was inserted. There were no major complications, and the patient was discharged home on postoperative day 23. Surgical details of the preemptive FET in this case are shown in the Video.

## Comment

**Figure 3:**
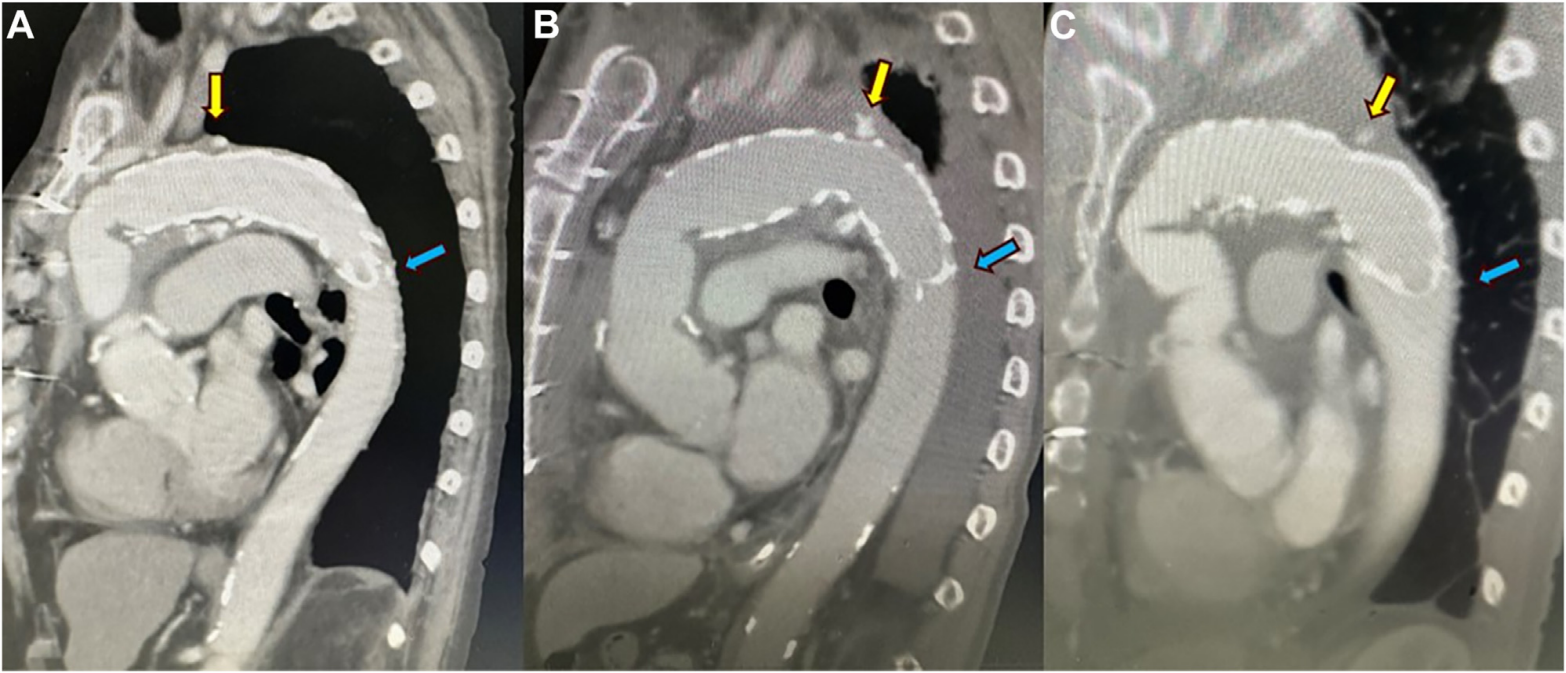
Postoperative computed tomography image of each patient. (A, B) Patients 1 and 2 with infectious aortic aneurysms. (C) Patient 3 with aortic coarctation. Yellow arrows indicate the distal anastomosis line. Blue arrows indicate the distal edge of the frozen elephant trunk.

Bleeding at the anastomosis site is a major concern in conventional total arch replacement.^1^ The risk increases in areas with limited working space and poor vision of the descending aorta. In these cases, hemostasis was achieved after sealing of the anastomosis site with the FET, and there was no resternotomy for bleeding. Postoperative computed tomography images of each case are shown in Figure 3. Although there is no comparable evidence for preemptive FET, we consider this to be a new method, with advantages of not only being able to handle deeper and more difficult anastomosis sites but also having less risk of collapse when anastomosing 3 artificial pieces—the straight artificial vessel, FET, and 4-branch artificial vessel.

The FET technique has a relatively high rate of paraplegia as a perioperative complication,^2^ with a higher probability than in thoracic endovascular aneurysm repair.^3^ Usui and colleagues^4^ suggested that interference of the FET with the blood supply to the spinal cord might contribute to spinal cord infarction. Preventza and coworkers^5^ reported that in type A acute aortic dissection, the incidence of paraplegia due to spinal cord injury was lower for a stent length of 10 cm than for 15 cm and for implantation beyond Th8. In these cases, the landing zone beyond the peripheral anastomosis was kept at 3 to 4 cm and retained more centrally than the Th8 level. As the FET can be inserted rapidly after peripheral single-tube anastomosis and peripheral blood supply by balloon is started immediately, the time required to stop the lower body circulation is negligible. Accordingly, no paraplegia occurred in any of our 3 cases.
